# Command economies, graduated responsibility, and Competence-Based Medical Education

**DOI:** 10.36834/cmej.69772

**Published:** 2020-09-23

**Authors:** Eric Prost

**Affiliations:** 1 Queen’s University, Ontario, Canada

## Abstract

Competence-Based Medical Education (CBME) rightly emphasizes that residents should actively take charge of their own education by ensuring they are progressing towards competence in an array of Entrustable Professional Activities (EPAs). Paradoxically, many CBME curricula then dictate exactly how this is to happen by listing a multitude of variables that must be checked off regarding the specifics of cases encountered. This is burdensome and unrealistic as well as contrary to the spirit of CBME. We want residents to know how to learn so they can problem solve in new situations. This is not achieved by dictating that they see nearly everything during their residency. Command economies with complete and rigid planning from above do not work. This also applies to residency training.

## Abstract (fr)

La formation médicale basée sur les compétences (FMBC) souligne à juste titre que les résidents devraient prendre activement en main leur éducation en veillant à développer leurs compétences dans une variété d’activités professionnelles confiables (APC). Paradoxalement, un grand nombre de programmes de FMBC dictent précisément comment cela devrait se passer en dressant la liste d’une multitude de variables qui doivent être cochées selon les particularités des situations cliniques rencontrées. Ceci est fastidieux et peu réaliste de même que contraire à l’esprit de la FMBC. Nous voulons que les résidents sachent comment apprendre de façon à ce qu’ils puissent résoudre les problèmes lorsqu’une nouvelle situation se présente. Il n’est pas possible d’arriver à ce but en exigeant qu’ils soient exposés à presque tout durant leur résidence. Les économies dirigées dont la planification complète et rigide imposée par la direction ne fonctionnent pas. Ceci s’applique aussi aux programmes de résidence.

The glory of Competence-Based Medical Education (CBME) is that it lets learners take charge of their own education, allowing them to have skills entrusted to them once they have mastered them rather than after a specified length of time or number of repetitions. CBME also puts the responsibility largely on the learners to plan their education and to take charge of ensuring they have seen enough patients to consolidate their learning. The assessments are usually triggered by the residents themselves. Rather than passively awaiting a report card at the end of a rotation, CBME residents must be constantly thinking about what experiences they have had and whether they are working towards the right Entrustable Professional Activities (EPAs), and at the right pace. They need to be the ones to send assessment requests to their supervisors in order to advance towards the entrustment of the necessary skills.

CBME is not based on time spent on a rotation. Rather than have long blocks and sheer volume to ensure certain types of cases are encountered (and mastered), CBME curricula often stipulate exactly how many of each type of patient or case must be seen in order to attain a certain EPA. For example, one activity might be to manage common psychiatric presentations in children and youth. Within this EPA there might then be multiple contextual variables that must be met such as a certain number of cases of depression, a certain number of patients younger than 12-years-old, at least four different assessors, at least three clinical settings, etc. This all makes some sense, but recently it has spiraled out of control. As the various specialties across Canada meet to design their own CBME curricula, it is easy to be rigidly prescriptive and to allow these contextual variables to proliferate and even overtake the process.

We must not burden residents. We are asking them to take charge of their education as adult learners and then, ironically, immediately dictating exactly what they must learn, right down to the specific number of times they must see and excel at often narrow learning scenarios. Is this realistic? Residents cannot control which patients walk into clinic while they are on rotations, so, in practice CBME has already necessitated residents returning to past rotations after they have moved on in order to see one or two patients with particular features or certain diagnoses.

We have long realized and then rightly emphasized that, because of the vast and dynamic nature of medical knowledge, a medical student cannot know everything at the end of four years. We also surely recognize that a resident cannot know all at the end of a five year residency. Therefore, we have helped learners to know how to know, how to learn, and where to find answers. We have guided them to ask the right questions rather than knowing definitively the correct—and, in many cases, soon obsolete—answer. Just as residents cannot know all at the end of residency, they cannot see all during residency.

As we begin CBME, our residency programs should also embed graduated responsibility as an integral feature. Years ago, a wise internist told our class that in medical school we were still students in the best sense of the word—still discovering, malleable, and often engaged in the theoretical—but that once we started residency, we would be participating in a hands-on practical endeavour. His point was that residency is an apprenticeship. And, in an apprenticeship, there is graduated responsibility, with more direction given at the early stages. This diminishes with emerging expertise, though, until supervision is minimal. Our residency programs should be organized in this way, ensuring that certain practices are honed, and core cases encountered, so that a learner cannot graduate without certain skills. However, any prescriptive planning must decrease as the resident progresses. And we are deceiving ourselves if we think learners can see all or can even tolerate the burden of trying.

Command economies with complete and rigid planning from above do not work. Residents are intelligent experienced learners, especially our senior residents; we should not dictate to them the minutiae of the curriculum. Mixed economies with some sensible planning and some reasonable freedoms do work. Helping residents learn how to learn, all the while enjoying themselves, rather than checking off compulsory boxes, is still valuable.

I admit this essay has limitations, since, like a tea bag, I have been steeping for far too long. Rather than dangling briefly in a cup of boiling water, letting my anti-oxidant properties escape for the benefit of all, I have been immersed in CBME for five full years. This allows me to write from some experience, but, like tea, whether green, black, or even herbal, after such a long immersion I have become a little bitter.

